# The role of microcircuits in the pre-frontal cortex in detecting and encoding temporally patterned information

**DOI:** 10.1186/1471-2202-16-S1-P197

**Published:** 2015-12-18

**Authors:** Constantinos Melachrinos, Athanasia Papoutsi, Panayiota Poirazi

**Affiliations:** 1 IMBB, FORTH, Heraklion, 70013, Greece

## 

Working memory is the capacity of the brain to hold information temporarily for immediate use. The neural correlate of this type of short-term memory is persistent spiking activity (PA) of both excitatory and inhibitory neurons, mainly in the pre-frontal cortex (PFC). Neurons embedded in PFC microcircuits integrate widespread information from various brain regions. PFC microcircuits of ~ten neurons are characterized by highly reciprocal connections and non-linear integration in their dendrites. Understanding how dendrites integrate information from multiple sources is crucial to explain their functional role. Spatial and temporal integration of signals occurs at dendrites before propagating to the soma, and plays a role in coding of information [1,2]. The main question is whether and how pyramidal neurons in such circuits can detect temporally structured information in order to timely adjust behavior. Dendrites of cortical pyramidal neurons have been shown to exhibit temporal sensitivity [1].

To address this question, we constructed a detailed PFC microcircuit, implemented in the NEURON simulation environment. We used reconstructed morphologies of layer 5 PFC pyramidal neurons, validated against experimental findings. The microcircuit consisted of nine pyramidal neurons and two interneurons, all interconnected [3]. To investigate temporal coding, we delivered temporally structured input to four out of the nine pyramidal neurons and assessed, given persistent activity emergence, a) the time-to-first-spike (ttfs) of each pyramidal cell and b) the Inter-Spike Intervals (ISIs) during PA.

We find that temporally patterned inputs (simulated as different temporal orders of activated neurons) induce different responses, in both the ttfs and ISI distributions. To investigate the mechanisms that mediate this type of coding, we varied both the stimulus frequency and the pyramidal neuron morphologies in the microcircuit. We found that lower stimulus frequencies resulted in increased differences between various temporal orders of activation. The same result was observed when using neurons with morphologically complex dendritic trees, indicating that dendritic morphology may play a key role in the ability of PFC microcircuits to detect and encode temporally patterned inputs. Further, we investigated whether this type of temporal coding exhibits *specificity*, i.e. whether the PFC can consistently interpret similar temporally patterned inputs in a spectrum of background activity environments.

Overall, this study seeks to understand at what level and how neuronal circuits implement the timely firing of PFC pyramidal neurons. Improving our understanding of temporal coding in complex areas like the PFC is essential for disentangling how the brain detects, encodes and transmits information. While dendrites of cortical neurons have been shown to detect differences in the order of incoming signals, our study breaks new ground in finding whether such a temporal code is *preserved *at the microcircuit level. These findings can be tested experimentally to further investigate the role of temporal coding in higher-level areas of the brain.
